# Speech Recognition for the iCub Platform

**DOI:** 10.3389/frobt.2018.00010

**Published:** 2018-02-12

**Authors:** Bertrand Higy, Alessio Mereta, Giorgio Metta, Leonardo Badino

**Affiliations:** ^1^iCub Facility, Istituto Italiano di Tecnologia, Genoa, Italy; ^2^Università di Genova, Genoa, Italy; ^3^Advanced Concepts Team, European Space Agency, Noordwijk, Netherlands; ^4^Center for Translational Neurophysiology of Speech and Communication, Istituto Italiano di Tecnologia, Ferrara, Italy

**Keywords:** automatic speech recognition, yarp, tensorflow, code:python, code:matlab, code:C++

## Abstract

This paper describes open source software (available at https://github.com/robotology/natural-speech) to build automatic speech recognition (ASR) systems and run them within the YARP platform. The toolkit is designed (i) to allow non-ASR experts to easily create their own ASR system and run it on iCub and (ii) to build deep learning-based models specifically addressing the main challenges an ASR system faces in the context of verbal human–iCub interactions. The toolkit mostly consists of Python, C++ code and shell scripts integrated in YARP. As additional contribution, a second codebase (written in Matlab) is provided for more expert ASR users who want to experiment with bio-inspired and developmental learning-inspired ASR systems. Specifically, we provide code for two distinct kinds of speech recognition: “articulatory” and “unsupervised” speech recognition. The first is largely inspired by influential neurobiological theories of speech perception which assume speech perception to be mediated by brain motor cortex activities. Our articulatory systems have been shown to outperform strong deep learning-based baselines. The second type of recognition systems, the “unsupervised” systems, do not use any supervised information (contrary to most ASR systems, including our articulatory systems). To some extent, they mimic an infant who has to discover the basic speech units of a language by herself. In addition, we provide resources consisting of pre-trained deep learning models for ASR, and a 2.5-h speech dataset of spoken commands, the VoCub dataset, which can be used to adapt an ASR system to the typical acoustic environments in which iCub operates.

## 1 Introduction

Several applications use speech to give instructions to iCub, often relying on proprietary software. However, the robot operates in specific conditions where those systems may perform poorly. An open and easy-to-use system that would reliably recognize commands in this context would thus be a very desirable tool. We present here a first codebase, henceforth *iCubRec*, which has been built to provide such services to the community of iCub users. It allows to train and run state-of-the-art deep neural network (DNN)-based automatic speech recognition (ASR).

As an additional contribution, a second codebase, henceforth *bioRec*, allows to experiment with novel DNN-based recognition systems that share the same bio-inspired and developmental learning view that gave birth to iCub (Lungarella et al., 2003). *bioRec* is self-contained and independent of *iCubRec*, however its DNN-based acoustic models can effortlessly be used within *iCubRec*.

Finally, in addition to the code, we are also providing resources to facilitate the implementation of a command recognizer: (i) the VoCub dataset, a dataset of registered vocal commands and (ii) pre-trained Gaussian Mixture Model (GMM)- and DNN-based acoustic models to perform recognition.

Our code, as well as the resources, is released under GPLv3 license. The code is available at https://github.com/robotology/natural-speech (doi: 10.5281/zenodo.1064043).

## 2 iCubRec

### 2.1 Application and Utility

An ASR system for iCub typically operates in challenging conditions. We have identified three specific factors which we want the system to be robust to:

- noise; the robot often operates in noisy environments (e.g., noisy servers and computers running, concurrent speakers, the robot itself generating noise).
- accents; the teams working with iCub are international and the robot needs to recognize spoken commands uttered with a wide variety of foreign accents.
- distance and movement; distant speech recognition is an important research topic in ASR and has been the focus of many recent challenges (e.g., the Chime4 challenge^1^). When the speaker–microphone distance increases, the speech signal-to-noise ratio decreases and signal distortions due to reverberation (in indoor environments) increases. A non-fixed distance, due to a moving speaker and/or microphone, adds further complexity to the task.

Although deep learning has recently produced excellent results in ASR, it still suffers the training-testing mismatched conditions problem. Proprietary ASR systems may perform poorly in the aforementioned acoustic/speech conditions mainly because such conditions are not well covered by their training datasets. We have addressed this problem by building a dataset (VoCub dataset) that covers such conditions and by providing tools to easily adapt a DNN to it.

Other than robust, an ASR system for iCub should be easy-to-use, open, and modular. Usability is necessary to allow all iCub mindware developers, who mostly have no ASR background, to train and run ASR on iCub. For this reason, we provide pre-trained GMM- and DNN-based acoustic models that can be used out of the box with the existing code. At the same time, we want more advanced users to easily modify and adapt the code to their own needs. This can only be done if everything is open and well modularized.

### 2.2 Methods

To facilitate the understanding of the *iCubRec* module for non-ASR experts we provide here the definition of few basic ASR terms. A standard ASR system consists of 4 main parts: an acoustic feature extraction step which extracts spectral features from the input acoustic waveform; an acoustic model which relates the extracted features to sub-words (e.g., phonemes, such as consonants and vowels) and then words (i.e., computes the likelihood that vectors of features are generated by a candidate word); a language model, which is independent of the acoustic signals and incorporates prior knowledge about a specific language (e.g., the probability that the word “barks” follows the word “dog”); and a speech decoder which performs word recognition by computing the most probable sequence of words of the utterance, given: (a) the acoustic model; (b) the language model; (c) the dictionary, which consists of all words the system has to recognize along with their phoneme transcriptions. Acoustic modeling is usually done using a Hidden Markov Model (HMM) which is well suited for sequential data like speech. HMMs combine transition probabilities (i.e., *p*(*s_t_* | *s*_*t*–1_) where *s_t_* is a phone label at time *t*) with observation probabilities (i.e., *p*(*o_t_* | *s_t_*), where *o_t_* is the input vector of acoustic feature at time *t*). The core difference between classical GMM-HMM vs. hybrid DNN-HMM acoustic models simply resides on whether GMMs or DNNs are used to compute the observation probabilities.

### 2.3 Code Description

iCubRec code is based on the Hidden Markov Model Toolkit (HTK) (Young et al., 2015). However, as the training capabilities for DNNs are still quite limited in HTK, we also consider the alternative possibility to train a network with Tensorflow (Abadi et al., 2015) and convert it to HTK format for use in decoding. Although in the later case the DNN is still restricted to the architectures recognized by HTK (for now, only feedforward networks with a limited set of activation functions), this gives more flexibility and control over the training process. Additionally, the use of Tensorflow allows to easily adapt a pre-trained DNN to new adaptation data.

The code consists of scripts for:

- acoustic model training with GMMs
- acoustic model training with DNNs
- speech decoding
- integration within YARP for online speech decoding.

iCubRec is a combination of Python 3, Perl and shell scripts, and was written for HTK 3.5 and Tensorflow 1.0.

#### 2.3.1 GMM-Based Acoustic Modeling

Before the advent of DNNs, GMM-HMM systems were state-of-the-art for acoustic modeling in speech recognition. Although they are significantly outperformed by neural networks (Dahl et al., 2012; Seltzer et al., 2013), GMMs are still widely used if only to compute the phone labels/speech segments alignments needed to train a DNN (Dahl et al., 2012). The folder gmm_training provides a set of scripts to train GMM-HMMs using HTK. These scripts are based on Keith Vertanen’s code (Vertanen, 2006) and allow to build models similar to the ones described by Woodland et al. (1994). The recipe is originally intended for TIMIT (Garofolo et al., 1993a) and Wall Street Journal (WSJ) (Garofolo et al., 1993b) datasets and has been adapted for the Chime4 challenge (Vincent et al., 2016) and VoCub datasets.

#### 2.3.2 DNN-Based Acoustic Modeling

Once the speech signal has been aligned (presumably using GMM-HMMs), a DNN-based model can be trained. Two alternatives are available: (i) using the scripts in dnn_training/htk to train a model with HTK or (ii) using the code under dnn_training/tf to train the net with Tensorflow. The scripts proposed here are currently restricted to TIMIT and WSJ, but support for additional datasets will be added soon.

#### 2.3.3 Speech Decoding

With a model trained with HTK (GMM-based or DNN-based), it is then straightforward to perform recognition on a new utterance. The folder offline_decoding provides an example of decoding on pre-recorded data with HTK. Additionally, export for_htk.py shows how to easily extract the parameters of a net trained with Tensorflow and convert them into HTK format.

#### 2.3.4 Integration with YARP

All the code presented so far is meant to train and test a system offline. yarp_decoding folder provides the modules necessary to use an existing model within YARP and perform online recognition. A streaming service based on yarp.js^2^ allows to record sound from any device equipped with a microphone and a web browser. Two other modules are provided: rctrld_yarphear_asr which saves the recorded data in a file, and the decoder (based on HVite tool from HTK) for feature extraction and command decoding. The application speechrec.xml is available to easily run and connect all the modules.

### 2.4 Resources

#### 2.4.1 The VoCub Dataset

Recording a dataset has two main advantages: (i) it allows to easily test the recognition system and to reliably estimate its performance in real conditions and (ii) can be used to adapt the system in order to reduce the training/testing mismatch problem. For this reasons, we have recorded examples of the commands we want to recognize within real-usage scenarios. That resulted in the VoCub dataset.^3^

The recordings consist of spoken English commands addressed to iCub. There are 103 unique commands (see Table 1 for some examples), composed of 62 different words. We recorded 29 speakers, 16 males and 13 females, 28 of them are non-native English speakers. We finally obtained 118 recordings from each speaker: of the 103 unique commands, 88 were recorded once, and 15 twice (corresponding to sentences containing rare words). This results in about 2 h and 30 min of recording in total.

**Table 1:** Examples of the commands used in the VoCub dataset.

I will teach you a new object.
This is an octopus.
What is this?
Let me show you how to reach the car with your left arm.
Let me show you how to reach the turtle with your right arm.
There you go.
Grasp the ladybug.
Where is the car?
No, here it is.
See you soon.

A split of the speakers into training, validation, and test sets is proposed with 21, 4, and 4 speakers per set, respectively. The files are organized with the following convention setid/spkrid/spkrid_cond_recid.wav, where:

- setid identifies the set: tr for training, dt for validation and et for testing.
- spkrid identifies the speaker: from 001 to 021 for training, 101 to 104 for validation and 201 to 204 for testing.
- cond identifies the condition (see below).
- recid identifies the record within the condition (starting from 0 and increasing).

The commands were recorded in two different conditions, a non-static (cond = 1) and a static condition (cond = 2), with an equal number of recorded utterances per condition.

In the static condition, the speaker sat in front of two screens where the sentences to read were displayed. In the non-static condition, the commands were provided to the subject verbally through a speech synthesis system, and the subject had to repeat them while performing a secondary manual task. This secondary task was designed to be simple enough to not impede the utterance repetition task, while requiring people to move around the robot. The distance between the speaker and the microphone in this last condition ranges from 50 cm to 3 m.

We also registered a set of additional sentences for the testing group (same structure but different vocabulary) to test the recognition system for new commands not seen during training. The sentences consist of 20 new commands, pronounced by each speaker of the test set twice: once in non-static condition (cond = 3) and once in static condition (cond = 4).

#### 2.4.2 Trained Models

As not all the datasets used in our scripts are freely available, and in order to ease the use of our system, we provide pre-trained acoustic models that can be used out of the box. The models/README.md file contains links to download GMM-based models trained on WSJ, Chime4 and VoCub datasets, and DNN-based models trained on TIMIT and WSJ. Additional DNN-based models will be added in the future. Further details about the different models and the precise training procedure can be found in the same file.

### 2.5 Example of Use

A good demonstration of the capabilities of the code presented so far is given in the file icubrec/DEMO.md. In a few simple steps, the user is shown how to perform offline decoding on the VoCub dataset with a pre-trained model. This example is accessible to novice ASR users and does not require any proprietary dataset.

A more in-depth example is given in icubrec/TUTORIAL.md, which provides detailed instruction on how to train a full ASR system on the WSJ dataset. This tutorial goes through all the main steps: training of a GMM-based acoustic model, computation of the alignments, training of a DNN-based acoustic model using those alignments, and finally decoding of the test sentences.

## 3 bioRec

### 3.1 Application and Utility

Our module for bio- and cognitive science-inspired ASR is composed of two distinct parts serving different purposes: *Articulatory Phone Recognition* and *Unsupervised/Developmental ASR*.

#### 3.1.1 Articulatory Phone Recognition

This part includes modules phonerec and pce_phonerec, which build *articulatory* phone recognition systems. A phone recognition system recognizes the sequence of phones of an utterance. It can roughly be identified as an ASR system without language model and dictionary. *Articulatory* phone recognition uses prior information about how the vocal tract moves when producing speech sounds. This *articulatory* view is strongly motivated by influential neurobiological theories of speech perception that assume a contribution of the brain motor cortex to speech perception (Pulvermüller and Fadiga, 2010) and have been shown to outperform strong DNN-based baselines where no prior articulatory information is used (see, e.g., Badino et al. (2016)).

#### 3.1.2 Unsupervised/Developmental ASR

The second part of *bioRec*, zerorchallenge, builds “unsupervised” ASR systems. Most recognition systems, including the *articulatory* systems, are trained on supervised data, where training utterances are associated to phonetic transcriptions, and the inventory of phones is given. This learning setting is far easier than the learning setting of an infant who has to acquire her native language and has to discover the basic units of the language on her own. In order to better understand how an infant can acquire the phone inventory during development from raw “unsupervised” utterances, we have created “unsupervised” ASR systems that were submitted and evaluated at the 1st Zero Resource Speech Challenge (ZRS challenge) (Versteegh et al., 2015).

### 3.2 Methods

#### 3.2.1 Articulatory Phone Recognition

**Figure 1:**
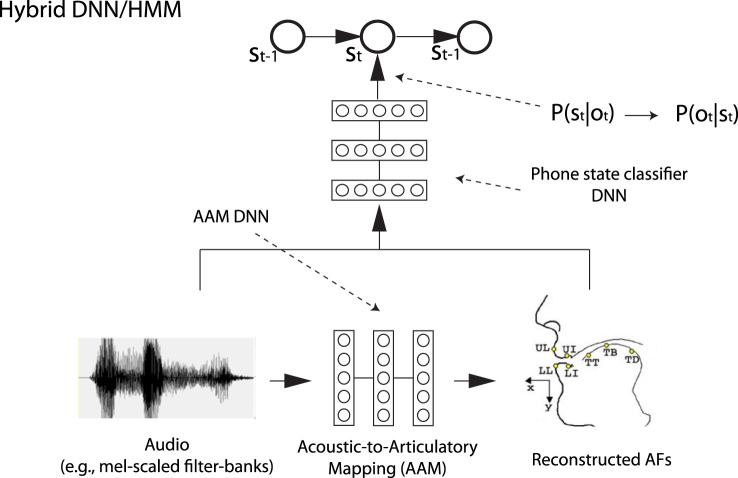
An example of articulatory phone recognition. Here, the simplest strategy available in phonerec is shown. o_t_ is a vector of acoustic features, while s_t_ is a phone state.

The *articulatory* phone recognition module consists of 2 parts depending on how speech production information is represented:

- phonerec; speech production is represented in the form of actual measurements of vocal tract movements, collected through instruments such as the electromagnetic articulograph (Richmond et al., 2011);
- pce_phonerec; vocal tract movements are initially described by discrete linguistic features and actual measurements are not used.

phonerec: in this module, prior information of speech production is built by learning, during training, an acoustic-to-articulatory mapping that allows to recover vocal tract movements, i.e., reconstructed articulatory features (AFs), from the acoustic signal (Badino et al., 2012, 2016). The reconstructed AFs are then appended to the usual input acoustic vector of the DNN that computes phone state posterior probabilities, i.e., the acoustic model DNN (see Figure 1, which shows the simplest strategy). Additionally, our code allows to apply autoencoder (AE)-based transformations to the original AFs in order to improve performance. AEs are a special kind of DNN that attempts to reconstruct its input after encoding it, typically through a lossy encoding. More details and evaluation results can be found in Badino et al. (2016).

pce_phonerec: in this module, AFs are derived (through a DNN) from linguistic discrete features (referred to as phonetic context embedding). They are used as secondary target for the acoustic model DNN within a multi-task learning (MTL) strategy (Caruana, 1997). This strategy forces the DNN to learn a motor representation without the need for time-consuming collection of actual articulatory data. Our approach outperforms strong alternative MTL-based approaches (Badino, 2016).

#### 3.2.2 Unsupervised/Developmental ASR

**Figure 2:**
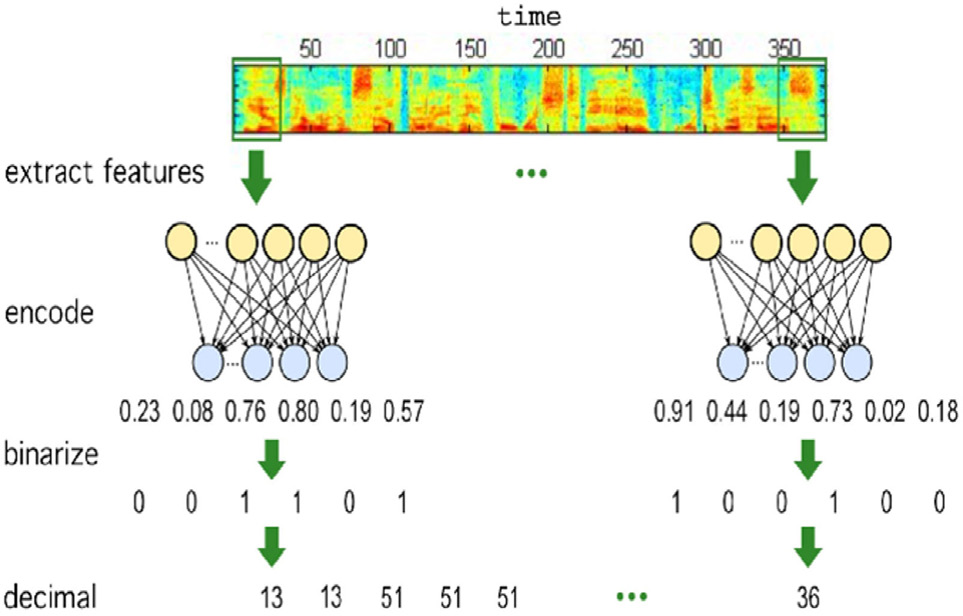
Overview of the AE-based approach to sub-word learning.

zerorchallenge is the module building the unsupervised/developmental ASR systems we submitted to Task1 of the ZRS challenge at Interspeech 2015 (Versteegh et al., 2015). The goal of the challenge was to compare systems that create new acoustic representations that can discriminate examples of minimal pairs, i.e., words differing only in one phoneme (e.g., “hat” vs. “had”), while identifying as a single entity different examples of a same word. Specifically, we focused on extracting discrete/symbolic representations, which equals to automatically discovering the inventory of (phone-like) sub-words of a language. Our core strategy is based on AEs (Badino et al., 2014), as shown in Figure 2. The provided scripts build 2 novel systems, one based on binarized AEs and one on Hidden Markov Model Encoders (HMM-Encoders) (Badino et al., 2015).

A binarized AE is an AE whose encoding layer nodes are binary. At each time step, it transforms a vector of real-valued acoustic features into a vector of binary units which in turn is associated to a positive integer corresponding to a discovered specific sub-word.

The HMM-Encoder combines an AE with a HMM.^4^ An approach solely based on AEs ignores the sequential nature of speech and inter-sub-word dependencies. The HMM-Encoder was proposed to specifically address these potential weaknesses.

### 3.3 Code Description and Example of Use

All code is written in Matlab and uses the Parallel Processing Toolbox to allow fast DNN training with GPUs. All modules were tested in Matlab 2013a and 2015a.

#### 3.3.1 Articulatory Phone Recognition

phonerec: the file ploclassify.m allows to train and test articulatory phone recognition systems. It requires the inivar.m configuration file where it is possible to define, e.g., the type of AFs through cmotortype (e.g., AE-transformed AFs or “plain” AFs), the hyperparameters of the acoustic model DNN (parnet_classifier), and of the acoustic-to-articulatory mapping DNN (parnet_regress).

The folder demo contains 2 examples to build and evaluate a baseline (audio1_motor0_rec0) and an articulatory phone recognition system (audio1_motor3_rec1) on the mngu0 dataset (Richmond et al., 2011). The dataset used here (available at https://zenodo.org/record/836692/files/bioRec_Resources.tar.gz, under/bioRec_Resources/phonerec_mngu0/) is a preprocessed version of the mngu0 dataset.

pce_phonerec: this articulatory phone recognition system is trained and evaluated by running mtkpr_pce.m. It can be compared with an alternative MTL-based strategy proposed by Microsoft researchers (Seltzer and Droppo, 2013), by running the script mtkpr_baseline.m. All systems are trained and tested on the TIMIT dataset, which unfortunately is not freely available. Training on different datasets would require some small dataset-dependent modifications to the look-up table used to extract discrete linguistic features from phone names.

We have created a Python + Tensorflow implementation the DNN training proposed in this module which will be soon available.

#### 3.3.2 Unsupervised/Developmental ASR

We provide scripts that receive as input one of the datasets provided by the ZRS challenge, train one of the unsupervised ASR systems (on the training utterances), and return the testing utterances in a new discrete representation with a positive integer at each time step. We additionally provide the 3 datasets from the ZRS challenge already transformed to be processed by our scripts (available at https://zenodo.org/record/836692/files/bioRec_Resources.tar.gz, under/bioRec_Resources/zerochallenge/). The output format allows to evaluate the output file with the tools provided for the challenge (Versteegh et al., 2015).

#### 3.3.3 Utilities

All utilities used by the phonerec, pce_phonerec, and zerorchallenge are in:

- netutils: contains functions to train and run DNNs, e.g., standard DNN training, Deep Belief Network-based DNN pretraining (Hinton et al., 2006), MTL training, DNN forward pass (i.e., to evaluate a DNN), deep autoencoder training, including training of some AEs we have recently proposed specifically for speech.
- utils: this folder contains all utilities that do not pertain to DNNs. These include: data loading and normalization, phone language models computation, Viterbi-based phone decoding, phone error rate computation, and analysis of error.

## 4 Conclusion

In this paper, we have described the codebase that allows to easily train deep neural network-based automatic speech recognition systems and run them within YARP. As an additional contribution, we provide tools to experiment with recognition systems that are inspired by recent influential theories of speech perception and with systems that partly mimic the learning setting of an infant who has to learn the basic speech units of a language.

## Ethics Statement

This study was carried out in accordance with the recommendations of the “Comitato Etico per la Sperimentazione con l’Essere Umano della ASL 3 di Genova” with written informed consent from all subjects. All subjects gave written informed consent in accordance with the Declaration of Helsinki. The protocol was approved by the “Comitato Etico per la Sperimentazione con l’Essere Umano della ASL 3 di Genova.”

## Author Contributions

Conceived and designed the ASR systems: LB, GM, BH, and AM. Wrote the code: LB, BH, and AM. Wrote the paper: BH and LB.

## Conflict of Interest Statement

The authors declare that the research was conducted in the absence of any commercial or financial relationships that could be construed as a potential conflict of interest. The handling editor declared a past co-authorship with one of the authors, GM.

## Footnotes

^1^http://spandh.dcs.shef.ac.uk/chime_challenge/chime2016/.

^2^https://github.com/robotology/yarp.js.

^3^Freely available at https://robotology.github.io/natural-speech/vocub/.

^4^Our HMM training code is a modified version of code from K. Murphy’s BayesianNet toolbox, available at https://github.com/bayesnet/bnt.
