# Supplementary material for: Speech Recognition for the iCub Platform
Source: Front Robot AI. 2018 Feb 12;5:10. doi: 10.3389/frobt.2018.00010 (PMC7805979; doi:10.3389/frobt.2018.00010)
Supplement: Supplementary file 1 [file Presentation_1.ZIP › natural-speech-master/icubrec/yarp_decoding/stream_audio/index.html]

YarpJS Stream Audio


Stream Audio

# Stream Audio

This simple application uses the Web APIs to send audio streams directly from the device microphone to the **YARP** network.
In particular it translates audio batches into Yarp::sig::Sound objects and then writes them on port **/soundStream/mic:o**
Furthermore it writes a Bottle through **/soundStream/trigger:o** upon clicking the streaming button in order to trigger the **yarpHear** module to start and stop saving to file.
